# Sex-specific disease modifiers in juvenile myoclonic epilepsy

**DOI:** 10.1038/s41598-022-06324-2

**Published:** 2022-02-21

**Authors:** Amy Shakeshaft, Naim Panjwani, Amber Collingwood, Holly Crudgington, Anna Hall, Danielle M. Andrade, Christoph P. Beier, Choong Yi Fong, Elena Gardella, Joanna Gesche, David A. Greenberg, Khalid Hamandi, Jeanette Koht, Kheng Seang Lim, Rikke S. Møller, Ching Ching Ng, Alessandro Orsini, Mark I. Rees, Guido Rubboli, Kaja K. Selmer, Pasquale Striano, Marte Syvertsen, Rhys H. Thomas, Jana Zarubova, Mark P. Richardson, Lisa J. Strug, Deb K. Pal

**Affiliations:** 1 Department of Basic and Clinical Neurosciences, Maurice Wohl Clinical Neurosciences Institute, Institute of Psychiatry, Psychology and Neuroscience, King’s College London, 5 Cutcombe Road, London, SE5 9RX UK; 2 MRC Centre for Neurodevelopmental Disorders, King’s College London, London, UK; 3 Program in Genetics and Genome Biology, The Hospital for Sick Children, 555 University Avenue, Toronto, ON Canada; 4 Adult Epilepsy Genetics Program, Krembil Research Institute, University of Toronto, Toronto, Canada; 5 Odense University Hospital, Odense, Denmark; 6 Division of Paediatric Neurology, Department of Paediatrics, Faculty of Medicine, University of Malaya, Kuala Lumpur, Malaysia; 7 Danish Epilepsy Centre, Dianalund, Denmark; 8 Nationwide Children’s Hospital, Columbus, OH USA; 9 Cardiff and Vale University Health Board, Cardiff, UK; 10 Department of Neurology, Oslo University Hospital, Oslo, Norway; 11 Division of Neurology, Department of Medicine, Faculty of Medicine, University of Malaya, Kuala Lumpur, Malaysia; 12 Department of Regional Health Services, University of Southern Denmark, Odense, Denmark; 13 Institute of Biological Sciences, Faculty of Science, University of Malaya, Kuala Lumpur, Malaysia; 14 Department of Clinical and Experimental Medicine, Pisa University Hospital, Pisa, Italy; 15 Neurology Research Group, Swansea University Medical School, Swansea, UK; 16 University of Copenhagen, Copenhagen, Denmark; 17 Division of Clinical Neuroscience, Department of Research and Innovation, Oslo University Hospital, Oslo, Norway; 18 National Centre for Epilepsy, Oslo University Hospital, Oslo, Norway; 19 IRCCS Istituto ‘G. Gaslini’, Genoa, Italy; 20 University of Genova, Genoa, Italy; 21 Department of Neurology, Drammen Hospital, Vestre Viken Health Trust, Oslo, Norway; 22 Newcastle Upon Tyne NHS Foundation Trust, Newcastle, UK; 23 Faculty of Medical Sciences, Translational and Clinical Research Institute, Newcastle University, Newcastle, UK; 24 Department of Neurology, Second Faculty of Medicine, Charles University and Motol University Hospital, Prague, Czech Republic; 25 King’s College Hospital, London, UK; 26 Departments of Statistical Sciences and Computer Science and Division of Biostatistics, The University of Toronto, Toronto, Canada; 27 Evelina London Children’s Hospital, London, UK

**Keywords:** Epilepsy, Prognosis

## Abstract

Juvenile myoclonic epilepsy (JME) is a common idiopathic generalised epilepsy with variable seizure prognosis and sex differences in disease presentation. Here, we investigate the combined epidemiology of sex, seizure types and precipitants, and their influence on prognosis in JME, through cross-sectional data collected by The Biology of Juvenile Myoclonic Epilepsy (BIOJUME) consortium. 765 individuals met strict inclusion criteria for JME (female:male, 1.8:1). 59% of females and 50% of males reported triggered seizures, and in females only, this was associated with experiencing absence seizures (OR = 2.0, *p* < 0.001). Absence seizures significantly predicted drug resistance in both males (OR = 3.0, *p* = 0.001) and females (OR = 3.0, *p* < 0.001) in univariate analysis. In multivariable analysis in females, catamenial seizures (OR = 14.7, *p* = 0.001), absence seizures (OR = 6.0, *p* < 0.001) and stress-precipitated seizures (OR = 5.3, *p* = 0.02) were associated with drug resistance, while a photoparoxysmal response predicted seizure freedom (OR = 0.47, *p* = 0.03). Females with both absence seizures and stress-related precipitants constitute the prognostic subgroup in JME with the highest prevalence of drug resistance (49%) compared to females with neither (15%) and males (29%), highlighting the unmet need for effective, targeted interventions for this subgroup. We propose a new prognostic stratification for JME and suggest a role for circuit-based risk of seizure control as an avenue for further investigation.

## Introduction

Juvenile myoclonic epilepsy (JME) is the most common idiopathic generalised epilepsy (IGE) syndrome^1^ with estimated prevalence of 5% to 10% of all epilepsies and 18% of IGEs^2^. It is a complex genetic disorder with likely polygenic inheritance but its genetic architecture, and environmental components of its aetiology, are currently unknown. Seizure prognosis varies, with 20–40% of patients never achieving seizure remission^3,4^, and has remained so for decades. We currently have insufficient evidence to predict which patients are at differential risk for poorer outcomes, or who may benefit from alternative treatment approaches. Such stratification is urgently necessary to improve patient outcomes.

Some prognostic factors are well-known in JME, others are less well-validated. A recent meta-analysis confirms that experiencing absence seizures is the strongest negative prognostic factor for seizure remission^4^. Seizure precipitants are frequently self-reported by patients with JME and other epilepsies^5–8^, with stress and sleep deprivation being the most commonly reported precipitants in JME^5,8^. Small case series suggest that certain, less common precipitants are associated with anti-seizure medication (ASM) resistance: for example, eye closure sensitivity^9^ and praxis-induced seizures^4,10^ in mixed-sex cohorts, and catamenial seizures in female cohorts^11^. These studies are insufficiently replicated and not adjusted for other potential confounders and, because of the high prevalence of self-reported precipitants, require additional investigation. Further, little is known about the influence of frequently reported stress-related precipitants on seizure control. Photosensitivity, defined here as either seizures triggered by light/visual stimuli and/or a photoparoxysmal response (PPR) evoked during an EEG, is also commonly reported but there are conflicting data about its prognostic significance^6,12,13^. Overall, the relationship between precipitants and seizure control is unclear and may also be confounded by sex differences.

Sex differences in JME presentation are pervasive. In addition to the overall female preponderance in JME^14,15^, females have a greater frequency of absence seizures^16^, triggered seizures, and photosensitivity^8,12,15,17^, suggesting important sex differences in both seizure susceptibility and cortical excitability^18–20^. Yet evidence for sex-specific prognosis in JME is conflicting^21^. Moreover, regulations governing valproate prescription in females of childbearing age^22^ result in systematic differences in ASM exposure between the sexes. These considerations motivate careful and detailed investigation of sex-stratified prognosis, which may give insights into sex-specific effects and the complex aetiology of JME.

Here, we introduce the Biology of Juvenile Myoclonic Epilepsy (BIOJUME) Consortium, an international study spanning 72 sites from 12 countries focused on young people and adults with JME. This is the world’s largest JME dataset and is uniquely rich in phenotypic depth and breadth, including demographic, clinical, behavioural, treatment and EEG data, thereby allowing in-depth analysis of prognostic factors through multivariable analysis. In this study, we aimed to investigate (i) the epidemiology of seizure precipitants in JME; (ii) the relationship between patient-reported precipitants and the objective EEG measured PPR; (iii) lifestyle interventions and their relationship with reported precipitants; and (iv) factors influencing seizure control, including sex, seizure types and precipitants. We hypothesize that modifiers of seizure control, such as absence seizures and seizure precipitants, will have sex-specific effects.

## Results

### General demographics and clinical features

864 individuals were reviewed by the phenotyping panel, and of these, 80 did not meet strict inclusion criteria for JME, leaving 784 eligible individuals. Reasons for ineligibility were an epilepsy syndrome other than JME (n = 52), ineligible age (n = 8), not enough clinical data to determine phenotype (n = 8), no EEG information available (n = 3), no evidence of interictal generalised spikes/polyspike and waves on EEG (n = 2), abnormal background EEG (n = 2), ineligible age of seizure onset (n = 2), dysmorphic features (n = 2) and learning disability (n = 1). Self-reported ancestry was 87% European, 9% Asian, 3% mixed ethnicity and the remaining 1% either African, Middle Eastern or Indigenous American. Demographic and clinical information for participants are displayed in Table 1.

**Table 1:** Demographics and clinical characteristics of the JME cohort.

	Male	Female	Total cohort
N	278 (36%)	487 (64%)	765 (100%)
Age (median, range) years	22 (9–53)	23 (6–53)	23 (6–53)
Age at myoclonic seizure onset (mean ± SD) years	14.7 ± 3.4	14.4 ± 3.2	14.5 ± 3.2
Absence seizures	98 (38%)	213 (45%)	311 (42%)
GTCS	241 (90%)	415 (88%)	656 (89%)
Self-reported triggered seizures	119 (50%)*	240 (59%)*	359 (56%)
Seizures triggered by stress-related precipitants	82 (34%)	168 (41%)	250 (39%)
Percentage of seizures triggered (median)	70%	63%	70%
Photosensitivity	78 (37%)**	206 (55%)**	284 (49%)
(Any) Response to photic stimulation during EEG	69 (29%)**	186 (44%)**	255 (38%)
Photoparoxysmal response	58 (28%)**	157 (42%)**	215 (37%)
Seizures triggered by photic stimulation	28 (12%)*	82 (20%)*	110 (17%)
Exclusively myoclonic seizures	9 (32%)	35 (43%)	44 (40%)
Myoclonic and other seizure types	6 (21%)	6 (7%)	12 (11%)
Other seizure types	3 (11%)	9 (11%)	12 (11%)
Unknown seizure type	10 (36%)	32 (39%)	42 (38%)
Seizure-free	127 (71%)	215 (66%)	342 (68%)
Drug-resistant	52 (29%)	113 (35%)	165 (33%)
Unknown/missing seizure control	99	159	258
History of valproate use	183 (66%)**	222 (46%)**	405 (53%)

For percentages, denominators are adjusted for missing data.

*Sex difference, *p* < 0.05.

**Sex difference, *p* < 0.001.

### Epidemiology of triggered seizures and photoparoxysmal response

We investigated the concordance of triggered seizures in genetically-related individuals with JME. There were 36 genetically-related individuals in the cohort, of whom 11 sibling-pairs (n = 22) and two sibling-trios (n = 6) were concordant for *not experiencing* triggered seizures; one sibling-pair and one parent–child pair were concordant for *experiencing* triggered seizures; one sibling-pair was discordant in their response; and one sibling-pair unknown. For all further analyses we included only one individual per family (included n = 17, removed n = 19) leaving 765 in the final cohort (Table 1).

In our final cohort, 56% of individuals reported having triggered seizures, with a significant female excess (OR = 1.4 (95% CI = 1.1–2.0), *p* = 0.02) (Table 1). Individuals with triggered seizures were older at the time of recruitment than those without (median 25 vs 21 years, U = 38,368, *p* < 0.001).

Next, we investigated whether specific seizure types are associated with experiencing triggered seizures. Absence seizures were experienced by 42% of participants. Having triggered seizures was significantly associated with experiencing absence seizures in females (OR = 2.0 (95% CI = 1.4–3.1), *p* < 0.001, N = 404), but this was not statistically significant in males (OR = 1.4 (95% CI = 0.84–2.5), *p* = 0.19, N = 233). Experiencing generalised tonic–clonic seizures (GTCS) was associated with experiencing triggered seizures in males (OR = 2.7 (95% CI = 1.1–6.8), *p* = 0.03, N = 237) but not statistically significant in females (OR = 1.5 (95% CI = 0.9–2.7), *p* = 0.16, N = 402). There was no association between age of myoclonic (U = 42,196, *p* = 0.98), absence (U = 4411, *p* = 0.57), nor GTC (U = 32,279, *p* = 0.76) seizure onset and experiencing triggered seizures.

PPR was documented in 37% of the cohort and was more common in females (OR = 1.9 (95% CI = 2.3–2.8), *p* < 0.001, Table 1). 17% of individuals reported a history of seizures triggered by intermittent photic stimulation (IPS), mostly myoclonic seizures (see Table 1). Other seizure types reportedly triggered by IPS included absences, GTCS or eyelid flickering/myoclonia.

### Epidemiology of seizure precipitants

The frequencies of reported seizure precipitants are shown in Table 2, with the most common being sleep deprivation. Females were more likely to report stress (OR = 1.6 (95% CI = 1.1–2.3), *p* = 0.01) and light/visual stimuli (OR = 1.7 (95% CI = 1.0–2.9), *p* = 0.04) as seizure precipitants than males, while more males reported playing games as a seizure precipitant (OR = 0.19 (95% CI = 0.1–0.7), *p* = 0.01). The majority of those reporting triggered seizures reported more than one precipitant, with 62% of precipitant-sensitive males (73/118) and females (147/239) reporting ≥ 2 precipitants.

**Table 2:** Frequency of reported seizure precipitants by sex and the most common importance ranking.

Trigger item	Frequencies					
	Males		Females		Total	
	N (%)	Rank	N (%)	Rank	N (%)	Rank
1. Sleep deprivation	75 (31)	1	147 (36)	1	222 (34)	1
2. Stress	52 (22)*	2	125 (31)*	1	177 (27)	1
3. Alcohol consumption	31 (13)	2	60 (15)	2	91 (14)	2
4. Light/visual patterns	22 (9)*	1	60 (15)*	1	82 (13)	1
5. Menstrual cycle	–	–	50 (12)	1	50 (8)	1
6. Concentration	14 (6)	3	22 (5)	3	36 (6)	3
7. Hunger/thirst	7 (3)	3	9 (2)	3	16 (3)	3
8. Speaking in public	3 (1)	4	10 (3)	3	13 (2)	3
9. Manipulation (praxis)	6 (3)	2	7 (2)	3	13 (2)	4
10. Playing games^a^	9 (4)*	4	3 (1)*	5	12 (2)	4
11. Calculation	3 (1)	3	3 (1)	2	6 (1)	3
12. Writing	1 (0)	4	4 (1)	2	5 (1)	2
13. Listening to music	2 (1)	3	1 (0)	3	3 (1)	3
14. Reading	1 (0)	6	2 (1)	6	3 (0.5)	6

^a^Note that this may have been interpreted as either playing physical sports or playing video games.

*Sex difference, *p* < 0.05.

We next explored whether absence seizures are associated with a sensitivity to specific precipitants in females (there was no seizure/precipitant association in males), and found associations with having seizures provoked by stress (OR = 2.2 (95% CI = 1.4–3.4), *p* < 0.001), menstrual cycle (OR = 2.5 (95% CI = 1.3–4.6), *p* = 0.004), sleep deprivation (OR = 1.7 (95% CI = 1.1–2.5), *p* = 0.01), alcohol (OR = 1.8 (95% CI = 1.0–3.1), *p* = 0.04) or speaking in public (OR = 11 (95% CI = 1.4–87.9), *p* = 0.007) (N = 404 for all analyses).

A subset of male and female participants (n = 142) estimated the percentage of their total seizures they felt were precipitated. The median percentage reported was 70%, with 18% (25/142) of individuals reporting that 100% of their seizures had been triggered. In this small subgroup, the most frequent precipitants reported were similar to the overall precipitant ranking (Table 2): sleep deprivation (84%), stress (56%), alcohol (44%), menstrual cycle (20%), light/visual patterns (20%), concentration (12%), playing games (12%), praxis (8%) and speaking in public (4%).

### Laboratory provocation and precipitants

We tested the association between self-reported triggered seizures and PPR, and observed a strong relationship between the two (OR = 2.8 (95% CI = 1.9–4.0), *p* < 0.001), with 71% (136/192) of those with PPR reporting triggered seizures. More specifically, 63% (46/73) of those who report light/visual patterns as a trigger also have PPR and 24% (46/192) of those with PPR report light/visual patterns as a trigger (OR = 3.9 (95% CI = 2.3–6.5), *p* < 0.001). The presence of PPR was associated to a lesser degree with other precipitants: stress (OR = 2.1 (95% CI = 1.2–3.3), *p* = 0.002), sleep deprivation (OR = 1.8 (95% CI = 1.1–2.8), *p* = 0.01), praxis (OR = 9.2 (95% CI = 1.1–77.3), *p* = 0.01) and concentration (OR = 3.1 (95% CI = 1.1–8.6), *p* = 0.02) in females (N = 349), and with alcohol (OR = 3.6 (95% CI = 1.5–8.6), *p* = 0.003, N = 202) in males.

### Lifestyle modifications and precipitants

In the full cohort, 496 (78%) participants reported lifestyle modifications had been advised or applied to either reduce risk from seizures or mitigate the triggering of seizures, and this proportion did not differ by sex (χ^2^(1) = 1.4, *p* = 0.24). The frequencies of different lifestyle modifications are presented in Table 3. The most frequent lifestyle modifications parallel the most frequent seizure precipitants. However, the frequencies of those reporting lifestyle modifications that address the specific precipitant they experience are somewhat low (Table 3), ranging from 65% (individuals reporting sleep deprivation as a precipitant being recommended/undertaking sleep hygiene) to 2% (individuals reporting catamenial seizures being recommended/undertaking menstrual management). There was no difference in the percentage of individuals being advised/applying lifestyle modifications in those with triggered seizures (273/345, 79%) compared to those without (207/266, 78%) (χ^2^(1) = 0.15, *p* = 0.70).

**Table 3:** Frequency of reported lifestyle interventions tabulated with reported seizure precipitants.

Lifestyle interventions	Total		Reported seizure precipitants											
			Sleep deprivation		Stress		Alcohol		Light/visual patterns		Menstrual cycle		Playing games^a^	
	N	Column %	N	Column %	N	Column %	N	Column %	N	Column %	N	Column %	N	Column %
Sleep hygiene	368	48	**143**	**64**	97	55	55	60	40	49	33	66	8	67
Stress reduction	221	29	74	33	**69**	**39**	21	23	27	33	26	52	8	67
Alcohol consumption advice	228	30	68	31	51	29	**47**	**52**	22	27	22	44	6	50
Avoiding certain light conditions	19	3	5	2	5	3	2	2	**6**	**7**	3	6	2	18
Adjusting diet	16	2	7	3	5	3	4	4	2	2	2	4	0	0
Modified screen exposure	8	1	1	1	1	1	1	1	**2**	**2**	1	2	**2**	**17**
Exercise adjustment	10	1	5	2	3	2	2	2	1	1	1	2	**1**	**8**
Water/swimming precautions	9	1	3	1	0	0	1	1	0	0	0	0	0	0
Driving regulations	8	1	3	1	1	1	1	1	0	0	0	0	0	0
Avoiding illicit drugs	5	1	1	1	1	1	0	0	0	0	0	0	0	0
Smoking cessation	4	1	4	2	3	2	1	1	1	1	0	0	1	8
Menstrual management	2	0	1	1	0	0	0	0	1	1	**1**	**2**	0	0

Frequency percentages given are the percent of individuals reporting that specific precipitant who are advised of the lifestyle intervention. Frequencies of lifestyle modifications relating to specific triggers are in bold.

^a^Note that this may have been interpreted as either playing physical sports or playing video games.

### Predictors of seizure control

**Figure 1:**
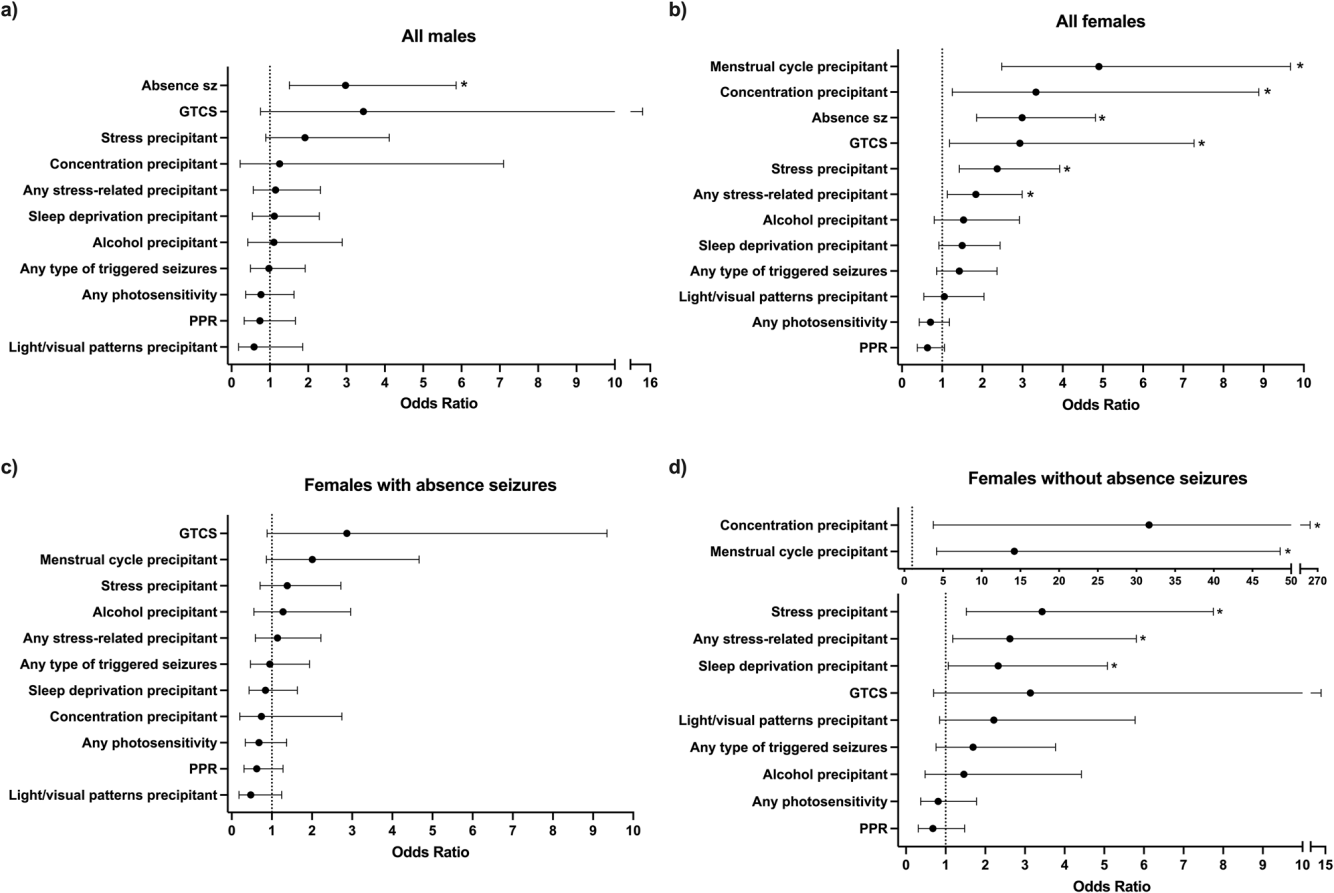
Predictors of drug resistance in subgroups of JME. Odds of being drug-resistant in males (**a**) and females (**b**) with seizure precipitant predictors (* = *p* < 0.05 in univariate analysis). Odds of drug resistance in females was further stratified by a history of absence seizures due to its association with triggered seizures in females (**c**, **d**). Note the two scales of x-axis (top and bottom) in (**d**).

We tested the association of seizure precipitants and other clinical variables with seizure control, using a dichotomous variable of drug resistance/seizure freedom in males and females (Fig. 1a, b, seizure triggers with low frequencies were not considered). There was no difference in overall prevalence of drug resistance between males and females (Table 1, χ^2^(1) = 1.5, *p* = 0.22). Drug-resistant individuals were significantly older at the time of recruitment than seizure-free individuals (median age seizure-free 22 years vs drug-resistant 26 years, U = 19,626, *p* < 0.001).

#### Seizure precipitants

In females, drug resistance was associated with experiencing seizures precipitated by menstrual cycle (OR = 4.9 (95% CI = 2.5–9.7), *p* < 0.001), stress (OR = 2.4 (95% CI = 1.4–3.9), *p* < 0.001) and concentration (OR = 3.3 (95% CI = 1.3–8.9), *p* = 0.012) in univariate analysis (N = 302). Experiencing PPR showed some indication of having a protective effect (better seizure control) in females (OR = 0.64 (95% CI = 0.4–1.1), *p* = 0.08, N = 269) (Fig. 1b). There was no association of any precipitant with seizure control in males (Fig. 1a). An increased number of seizure precipitants reported was associated with drug resistance in females (U = 8357, *p* = 0.02) but not males (U = 2661, *p* = 0.80). Reporting lifestyle modifications had no association with seizure outcome in males (OR = 0.8 (95% CI = 0.4–1.6), *p* = 0.49, N = 166) or females (OR = 1.1 (95% CI = 0.6–2.0), *p* = 0.73, N = 292).

#### General seizure characteristics

Drug resistance was highly associated with having absence seizures in both males (OR = 3.0 (95% CI = 1.5–5.9), *p* = 0.001, N = 173) and females (OR = 3.0 (95% CI = 1.9–4.9), *p* =  < 0.001, N = 325) (Fig. 1a,b). Drug resistance was also associated with having GTCS (OR = 2.9 (95% CI = 1.2–7.3), *p* = 0.016, N = 324) (Fig. 1b) and earlier myoclonus onset age (*p* < 0.001) in females (age of myoclonus onset < 12 years increases risk of drug resistance (OR = 2.0 (95% CI = 1.1–3.6), *p* = 0.031, N = 297)).

#### Do absence seizures modify the influence of seizure precipitants on seizure control?

**Figure 2:**
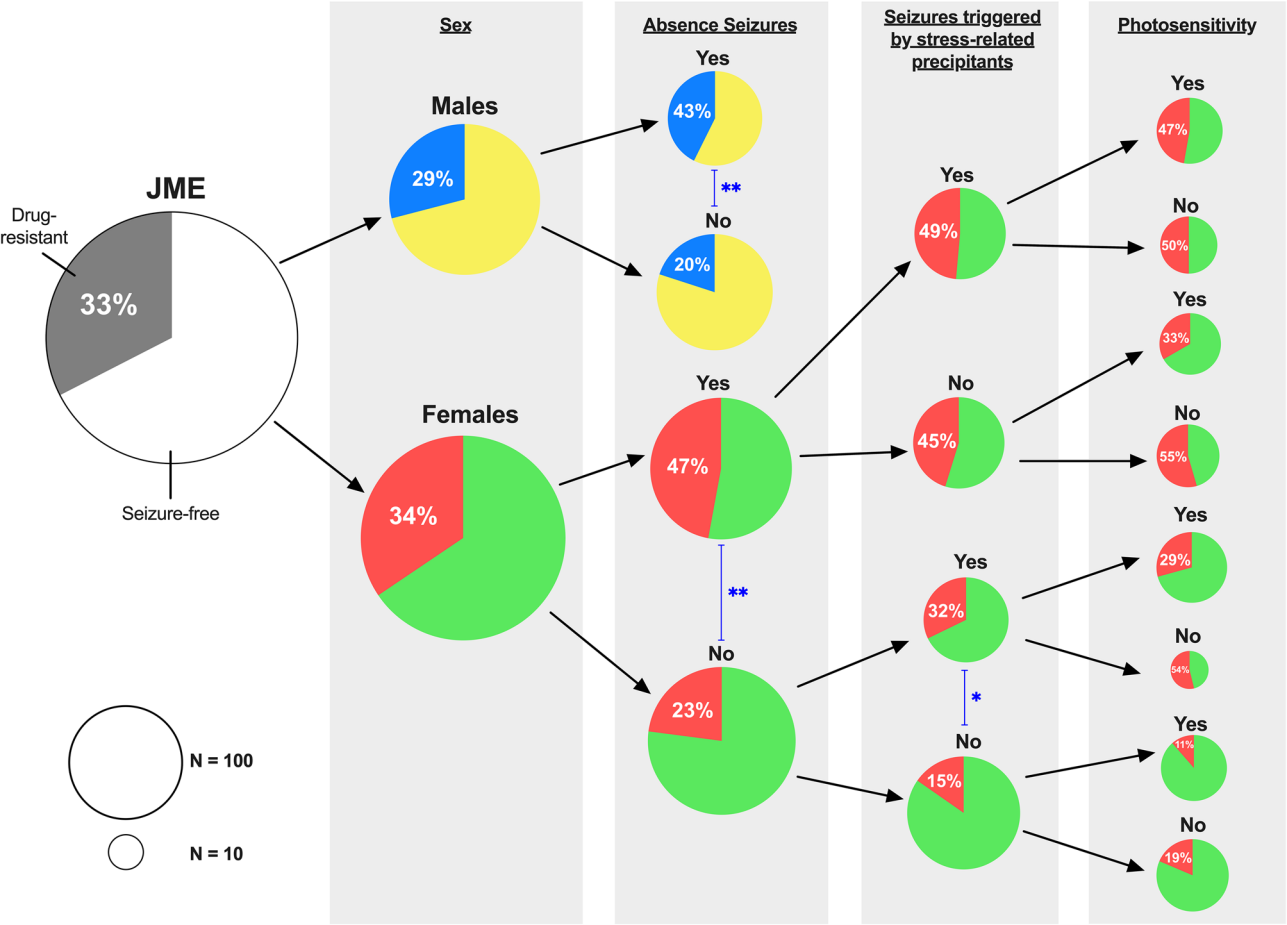
Differential rates of drug resistance in subgroups of JME. The right (white/green/yellow) segment of pie charts represent those seizure-free for over one year, and the left (grey/red/blue) segment represent those who are drug-resistant. Absence seizures moderate the effect of seizures triggered by stress precipitants in females, whereas photosensitivity (here defined as any response to photic stimulation during an EEG and/or reporting light/visual patterns as a trigger) is a protective factor for seizure control in all female strata. The area of the circle is proportional to the size of the subgroup in our cohort. **p < 0.01 and *p < 0.05 in chi-squared tests.

Above, we saw that triggered seizures are associated with absence seizures in females, and absence seizures are associated with drug resistance, therefore we further investigated the association of precipitants on drug resistance, stratified by absence seizures (Fig. 1c,d). Consequently, we found a marked difference in precipitant/seizure control associations, depending on whether females experience absence seizures. In females *without* absence seizures (N = 157), drug resistance was associated with experiencing seizures precipitated by concentration (OR = 31.6 (95% CI = 3.7–267.9), *p* =  < 0.001), menstrual cycle (OR = 14.2 (95% CI = 4.2–48.6), *p* < 0.001), stress (OR = 3.4 (95% CI = 1.0–11.8), *p* = 0.002) and sleep deprivation (OR = 2.3 (95% CI = 1.1–5.1), *p* = 0.03) (Fig. 1d). In females *with* absence seizures (N = 143) no seizure precipitants were associated with drug resistance (Fig. 1c). Stratifying females based on absence seizures and susceptibility to stress-related precipitants (concentration, stress, sleep deprivation or menstrual cycle), we see that only 15% (15/98) of those *without* absence seizures *or* susceptibility to stress-related precipitants are drug-resistant compared to 49% (34/70) of those *with both* absence seizures *and* stress-related precipitants. A summary prognostic stratification based on these results is illustrated in Fig. 2.

#### Multivariable analysis of seizure characteristics and precipitants on seizure control

Since experiencing absence seizures was the only variable associated with drug resistance in males, no multivariable analysis was carried out in males. We performed a logistic regression of drug resistance/seizure freedom including univariately associated precipitants in females (excluding concentration due to low frequency), as well as adjusting for other variables associated with seizure control (absence seizures, myoclonus onset age, GTCS and current age) (Table 4). In this model, absence seizures most significantly predicted poorer seizure control (OR = 6.0, *p* < 0.001), followed by having catamenial seizures (OR = 14.7, *p* = 0.001) and seizures precipitated by stress (OR = 5.3, *p* = 0.02). Effect modification of menstrual cycle precipitant on seizure control by absence seizures (*p* = 0.02) supported that catamenial seizures are only a predictor of poor seizure control in females without absence seizures. In contrast, PPR was associated with better seizure control (*p* = 0.03). A younger myoclonus onset age was also associated with drug resistance (*p* = 0.04).

**Table 4:** Multivariable logistic regression of drug resistance in females (N = 226).

Seizure-free/drug-resistant	OR (95%CI)	Coeff	Std. Err	Z	*p* value
(Intercept)	–	− 1.68	1.01	− 1.66	0.10
Absence sz	**5.97 (2.55**–**13.95)**	**1.79**	**0.43**	**4.13**	**0.000037**
Menstrual cycle precipitant	**14.71 (2.82**–**76.70)**	**2.69**	**0.84**	**3.19**	**0.0014**
Menstrual cycle precipitant*absence sz	**0.07 (0.01**–**0.61)**	− **2.64**	**1.09**	− **2.41**	**0.016**
Stress precipitant	**5.31 (1.32**–**21.39)**	**1.67**	**0.71**	**2.35**	**0.019**
PPR	**0.47 (0.24**–**0.94)**	− **0.75**	**0.35**	− **2.15**	**0.031**
Myoclonus onset age	**0.89 (0.80**–**0.99)**	− **0.12**	**0.06**	− **2.06**	**0.039**
GTCS	2.91 (0.94–9.03)	1.07	0.58	1.85	0.06
Current age	1.03 (0.99–1.07)	0.03	0.02	1.63	0.10
Sleep deprivation precipitant	0.41 (0.09–1.82)	− 0.88	0.75	− 1.17	0.24
Sleep deprivation precipitant*absence sz	0.49 (0.06–3.81)	− 0.72	1.05	− 0.68	0.49
Stress precipitant*absence sz	0.86 (0.12–6.23)	− 0.16	1.01	− 0.15	0.88

Seizure-free coded 0, drug resistant coded 1. OR = Odds ratio; sz = seizure; coeff = coefficient.

Bold *p* values are < 0.05.

*Indicates interaction terms.

#### The influence of valproate exposure on sex differences in seizure precipitants and seizure control

To investigate whether valproate efficacy is modulated by the presence of PPR, we stratified males based on the presence of PPR and history of valproate use. There was no difference in the frequency of drug-resistant individuals in these subgroups (χ^2^(3) = 1.5, *p* = 0.7, N = 136). Further, there was no association of any seizure precipitants on seizure control in a stratified group of males without a history of valproate use (N = 33, *p* > 0.3 for all precipitants) indicating that decreased valproate use in females compared to males (OR = 0.44 (95% CI = 0.32–0.59), *p* < 0.001, Table 1) is not responsible for sex differences we observe relating to seizure precipitants and seizure control.

## Discussion

BIOJUME constitutes the largest, most comprehensively phenotyped JME-specific cohort to our knowledge, allowing us to carefully address questions about prognosis not possible with smaller or less well-characterized datasets. Our results demonstrate evidence for sex-stratified seizure prognosis in JME. The sex difference in prognosis mediated by precipitants suggests a novel concept in IGE of disease modification by stress-related mechanisms in females. We further propose a new evidence-based stratification scheme for clinicians to focus management according to prognosis and risk factors. Using this scheme, we observe a three-fold increase in ASM resistance in females with *both* absence seizures *and* seizures triggered by stress-related precipitants (49%) compared to females with neither (15%). Finally, we show that photosensitivity has a positive effect on seizure control not explained by valproate therapy, suggesting a subgroup whose aetiology is addressed by current treatment.

Seizure precipitants are not unique to epilepsy but common in other episodic or cyclical neurological disorders like migraine, in which there is also a female bias in disease prevalence, severity, the frequency of precipitants and transformation to chronic disease^23^. In epilepsy, precipitants are reported more commonly in JME than other IGE syndromes or focal epilepsies^12,13^. In this study, around half of all individuals with JME report triggered seizures, with a significant female excess (59% vs 50%). Moreover, this subgroup reports a large proportion (median 70%) of their seizures are triggered, with 1 in 5 estimating that *all* their seizures are precipitated. This has immediate clinical relevance since recommended lifestyle modifications do not align with individual seizure precipitants. PPR and photosensitivity are also more common in females, consistent with previous studies showing a female excess of 1.5–2^8,17,24^. The female excess in PPR, triggered seizures and IGEs in general, implicates the influence of sex and steroid hormones, such as progestogens, androgens and oestrogens, on seizure susceptibility. A transcranial magnetic stimulation study in patients with JME showed differing patterns of fluctuating cortical excitability in females compared to males^20^, with studies in healthy controls indicating that the menstrual cycle contributes to these fluctuations^25,26^. The concordance for seizure precipitants among related individuals observed in this study suggests a genetic contribution to precipitant sensitivity.

Despite our exhaustive list, just five precipitants accounted for > 80% of the total: sleep deprivation, stress, alcohol, visual/lights and menstrual cycle, which are well-known in JME^5,8^ and other epilepsies^6,7,27^. Precipitants group according to two main neural circuits. First, stress-related precipitants: physiological states which influence neurobiological stress circuits, including stress itself, sleep deprivation, menstrual cycle, concentration and alcohol. Second, visual precipitants affecting circuits involved in visual/photosensitivity^28–30^, including both self-report and IPS; these were strongly associated, suggesting self-report represents a proxy measure of visual/photosensitivity.

Identifying risk factors for ASM resistance may inform alternate therapeutic approaches. The value of stratification is clear in this study where the overall sex difference in ASM resistance is non-significant, which likely contributes to inconsistent evidence for differential prognosis in females in previous studies^21^. In our study, we confirm that absence seizures are a strong independent risk factor for ASM resistance in both males (the only factor) and females. However, only in females do precipitants modify the odds of ASM resistance, depending on the presence of absence seizures. These differential odds are illustrated in Fig. 2. Briefly, stress-related precipitants negatively modify the odds of ASM resistance only in females *without* absence seizures; whilst visual/photosensitivity positively modifies the odds of ASM resistance in females *regardless* of absence seizures. While these seizure precipitants have been previously reported in females^11^, neither their sex-specific distribution nor their association with seizure control had been appreciated. Therefore, this is the first suggestion of environmental disease modification in IGE. A prospective longitudinal study would confirm the direction of the association between seizure precipitants and drug resistance, although there is no evidence to suggest that patients develop sensitivity to stress precipitants *after* failing to achieve seizure control^31^. Therefore, we hypothesise a differing genetic predisposition in females who are sensitive to stress-related precipitants, and that current ASMs do not address this susceptibility.

Individuals perceive and respond to stressors differently and can experience persistent sequelae depending on their level of stress resilience/vulnerability^32^. Brain anatomical and functional connectivity is an important determinant of individual stress resilience/vulnerability and is influenced by neurochemical and anatomical circuits including the neuroendocrine system (hypothalamic–pituitary–adrenal (HPA) axis), hippocampal pathways, the dopaminergic and serotonergic systems and medial prefrontal cortex (mPFC)^32,33^. Sexual dimorphism in stress susceptibility is linked to differential HPA axis activity via multiple neuroendocrine pathways^32^. Stress can alter structure and function in the prefrontal cortex^34^ while the mPFC also exerts strong negative control over stress pathways and mPFC lesions augment HPA axis response to emotional stress^35^. Previous studies indicate that individuals with stress-sensitive seizures exhibit blunted cortisol responses to acute stressors^36^ and have differential interactions of cortisol with brain functional connectivity^37^.

This novel understanding of prognostic classification (Fig. 2) focuses clinical and research implications onto: (i) females with stress-related precipitants, and (ii) more effective treatment of absence seizures. Almost half of females (49%) with both stress precipitants and absence seizures are ASM resistant, compared to 15% with neither. The overall figure of 33% ASM resistance conceals the large subgroup of females who do not respond to current ASMs. Future clinical trials should therefore consider stratifying participants with absence seizures and seizure precipitants. Also, stress-related precipitants should be routinely elicited in females. Our data show that an exhaustive list is superfluous—menstrual cycle, stress, sleep deprivation, concentration and visual sensitivity are sufficient to capture potential disease-modifying factors.

With the suggestion that sensitivity to stress-related precipitants may have a disease-modifying effect, we turn to data about lifestyle advice and interventions. We find a mismatch between the lifestyle advice given to patients and their reported precipitants, with an exceedingly small proportion of patients being recommended modifications to help with the seizure precipitants relevant to them (Table 3). This is especially true for catamenial seizures, which are the strongest independent risk factor for ASM resistance. We also found that lifestyle advice was not associated with seizure outcome, suggesting that current approaches are not effective in modifying disease course in females with precipitant-sensitive seizures. Overall, this indicates large gaps in applying targeted, evidence-based interventions for stress-related factors. Pharmacological and non-pharmacological interventions such as cognitive behavioural therapy, relaxation training, biofeedback and exercise should be evaluated^38^.

We also need to understand how visual pathway hypersensitivity acts as a protective factor in favour of seizure freedom, preferentially in females. Our finding suggests that failure to conduct a sex-stratified analysis explains the lack of overall association in a recent meta-analysis that found a protective effect of PPR on seizure freedom in four out of five studies^4^. One possibility is that the component of seizure susceptibility mediated via visual pathway hypersensitivity^28–30^ is effectively treated by current ASMs, a hypothesis we are unable to test in this study but merits further investigation.

The strong effect of absence seizures on ASM resistance, replicated multiple times^4^, and its strong association with trait impulsivity in JME^39^, mandates new thinking. Absence seizures are initiated by a distinct cortical network (involving occipital, prefrontal, precuneus, and medial parietal cortices^40^) from those involved in myoclonic seizures (prefrontal and motor cortices^41,42^ and cortico-striatal networks^42,43^), visual hypersensitivity^28–30^ and stress resilience^32,37^; however, propagation of activity across networks may explain seizure precipitation. Myoclonic and absence seizure types also have distinct genetic influences^44,45^. If absence seizures, visual sensitivity and stress-related precipitants in JME are not just clinical features but the instantiation of separate seizure susceptibility networks with their own distinct effect on seizure and behavioural outcomes^46^, then logically we should consider circuit-specific therapy^47^.

A cross-sectional study design with data collection from specialist clinics inevitably introduces ascertainment bias, as evidenced by a higher median age of drug-resistant individuals compared to seizure-free individuals, likely due to longer follow-up in clinics for those without seizure remission. A similar inflation in individuals reporting precipitants likely reflects their increased risk for drug resistance. However, we attempted to attenuate this ascertainment bias by including age in multivariable analyses. Self-reporting of seizures and precipitants is a common limitation in epilepsy studies. The utility of self-reporting seizure precipitants has previously been debated due to poor seizure-awareness and factors influencing self-perception of seizures, such as psychological state^27^. However, this study supports the credibility of self-perception of seizure precipitants due to their correlation with PPR, an objective EEG phenotype. There is also the possibility of attribution bias in recalling precipitants, which would falsely increase an association. Further, there may be subjectivity with participants/clinicians reporting seizure freedom, as we observed some individuals classified in this manner still reported infrequent myoclonic or absence seizures, as well as issues of medication non-compliance, which was not assessed here, contributing to a lack of seizure remission. Our assessment of lifestyle modifications was limited, and certain precipitants were only superficially characterised e.g., acute vs chronic stress, or the exact phase of menstrual cycle, were not distinguished. We did not assess comorbid psychiatric disorders and therefore could not include them in a multivariable model.

To conclude, there is a wide-ranging variability in prognosis between males and females with or without absence seizures in JME. Stress-related precipitants negatively modify the risk of ASM resistance in females, while visual/photosensitivity favours seizure freedom. Stratification reveals a lack of efficacy of current therapeutic approaches for large subgroups of females. Differential and complex neural stress responses may underlie this variability, which merit further research.

## Methods

### Participants and data collection

We collected cross-sectional data through the ongoing BIOJUME consortium study. Data for this study came from 864 individuals recruited retrospectively or prospectively from 58 sites across nine countries (Supplementary material). Inclusion criteria for this study are based on Avignon Class II consensus criteria for the diagnosis of JME^48^: (i) age of myoclonus onset 6–25 years; (ii) seizures comprising predominant or exclusive early morning myoclonus of upper extremities; (iii) EEG interictal generalised spikes/polyspike and waves with normal background. Participants aged between 6–55 years old were included. Exclusion criteria were: (i) myoclonus only associated with carbamazepine or lamotrigine therapy; (ii) EEG showing predominant focal interictal epileptiform discharges or abnormal background; (iii) any evidence of progressive or symptomatic myoclonus epilepsy or focal seizures; (iv) global learning disability; (v) dysmorphic features; (vi) unable to provide informed consent. We collected clinical data face-to-face through a structured questionnaire, augmented by clinical records and EEG reports. The dataset included general demographics and health information, epilepsy history, including seizure types, seizure frequency and drug/lifestyle interventions (Table 1).

### Clinical data assurance

Sites uploaded clinical data onto a secure central REDCap (Research Electronic Data Capture) database^49^ and study coordinators ensured the most complete and accurate data possible for each participant through iterative feedback. A phenotyping panel, comprising seven epilepsy experts (CPB, KH, DKP, MR, GR, MS, RT), evaluated the diagnosis of JME according to inclusion criteria, through consensus where necessary. Missing data exist for some retrospective cases where certain clinical details were unknown.

### Seizure precipitants

We compiled a list of seizure precipitants from the epilepsy literature^5–7^ and asked participants to answer in a binary response whether they identified any items from the list as precipitants of (myoclonic, absence or GTC) seizures. We then asked participants to rank the checked items in order of importance, with “1” being the most significant precipitant for that individual. We added a free field, “other”, to allow participants to include other precipitants not covered in the list. After data cleaning, we created a final list of 14 precipitants excluding null fields and including frequently mentioned items in the “other” field. For further analyses, we reduced the list to the most commonly reported items (frequency ≥ 2%) and grouped stress, menstrual cycle, concentration and sleep deprivation precipitants as stress-related precipitants. The occurrence of seizures or PPR provoked by intermittent photic stimulation (IPS) during an EEG recording was also reported, augmented from participant EEG reports. PPR was defined according to criteria in Kasteleijn-Nost Trenite et al.^50^. We operationally defined photosensitivity as anyone self-reporting seizures precipitated by light/visual patterns and/or PPR or seizures provoked by IPS.

### Lifestyle modifications

Participants and researchers reported whether lifestyle modifications had been advised or applied to either reduce risk from seizures, or mitigate the triggering of seizures, as well as a free field to report specific interventions. We categorized reported interventions into (i) sleep hygiene, (ii) alcohol consumption advice, (iii) stress reduction, (iv) avoiding certain light conditions, (v) adjusting diet, (vi) modified screen exposure, (vii) exercise modification (including both exercise promotion and exercise precautions), (viii) driving regulations, (ix) water/swimming precautions, (x) avoiding psychoactive drugs, (xi) smoking cessation, and (xii) menstrual management.

### Seizure control

To test associations of seizure precipitants with seizure control, we categorized participants based on their answers to two questions: (i) whether they had been free from seizures over the past year and (ii) current ASM therapy, categorized as either no drug therapy, monotherapy (not necessarily the first appropriate ASM), dual therapy, or drug-resistant (two or more ASM failures). Based on answers to these two questions, participants were categorized as either:

(i) Seizure-free, defined as those who have not had a seizure of any type in over a year (whether on no drug therapy, monotherapy or dual therapy) (N = 351, 45%) or,
(ii) Drug-resistant (either as reported or those who are not seizure-free on ≥ 2 ASMs) (N = 166, 21%).

Using this binary classification, 268 individuals were unable to be categorized, due to not fitting into either category (N = 129, 16%) or missing data (N = 139, 18%).

### Analysis procedure and statistical methods

We carried out statistical analysis in SPSS^51^ and STATA^52^ software and produced graphics on GraphPad Prism^53^. Prior to statistical testing, we checked for violation of test assumptions and chose statistical tests accordingly. *p* < 0.05 was used to determine significance. Categorical variables were compared using a Chi-squared test, or a Fisher’s exact test if expected frequencies were less than five. Missing data were excluded from each analysis in a pairwise manner.

To investigate predictors of seizure control, we first performed univariate analysis stratified by sex, followed by post-hoc stratification by absence seizures. Odds ratios (OR) and 95% confidence intervals (CIs) of the association of seizure precipitants and other clinical variables with drug resistance were calculated. Continuous and ordinal variables were tested for associations with seizure outcome using Mann–Whitney tests. Based on results from univariate analysis, logistic regression analysis was carried out for females, with significantly associated variables (seizure precipitants: menstrual cycle, stress and sleep; PPR; age of myoclonus onset; current age; generalised tonic–clonic seizures (GTCS); absence seizures) tested against the seizure freedom/drug resistance outcome.

To ensure results were not confounded by sex differences in valproate prescribing, we stratified males into four categories based on the presence of PPR and valproate use (since it is more frequently used in males and is effective in controlling seizures in photosensitive individuals^24^) and tested for any association with drug resistance. Further, we tested for associations of any precipitant variable with drug resistance in the sub-group of males without a history of valproate use.

### Study approval

BIOJUME is funded by the Canadian Institutes of Health Research (MOP-142405, FRN-167282) and received ethical approval from the National Health Service (NHS) Health Research Authority (South Central-Oxford C Research Ethics Committee, reference 16/SC/0266) and the Research Ethics Board of the Hospital for Sick Children, Toronto (REB#1000033784). Local ethical approvals were also held for all international sites. All procedures complied with appropriate regulatory requirements and ethical principles in line with the Declaration of Helsinki. Informed consent was obtained and documented for all participants. Assent was obtained from minors (under 16), and informed consent was obtained on their behalf by a parent or legally appropriate guardian. All clinical data from participants were de-identified before entry into the central database.

## Supplementary Information

Supplementary Information.

## Data Availability

Data supporting the findings of this study are available from the corresponding author upon reasonable request*.*
